# MEDIATING EFFECTS OF QUALITY OF LIFE IN THE ASSOCIATION OF DEBTS AND LATER MEMORY FUNCTION IN CHINA, ENGLAND, AND THE US

**DOI:** 10.1093/geroni/igad104.2425

**Published:** 2023-12-21

**Authors:** Tsai-Chin Cho, HwaJung Choi, Kenneth Langa, Sara Adar, Lindsay Kobayashi

**Affiliations:** University of Michigan School of Public Health, Ann Arbor, Michigan, United States; University of Michigan, Ann Arbor, Michigan, United States; University of Michigan, Ann Arbor, Michigan, United States; University of Michigan School of Public Health, Ann Arbor, Michigan, United States; University of Michigan School of Public Health, Ann Arbor, Michigan, United States

## Abstract

Non-mortgage debts were linked to negative cognitive health outcomes in later-life older adults. It is unclear how they may be associated with later-life memory function through psychosocial pathways, and whether the associations vary by health care system and policy environment. We examined the mediating effects of depressive symptoms and dissatisfaction with life in the association between non-mortgage debts and subsequent memory function among adults aged 65-101 years in the US, China, and England. Data were from harmonized, nationally representative longitudinal studies of aging in the US (Health and Retirement Study; n=8,388), China (China Health and Retirement Longitudinal Study; n=1,180), and England (English Longitudinal Study of Aging; n=3,390). Non-mortgage debt burden was defined as any pre-existing or new non-mortgage debts in two years (2010-2012 for the US and England; 2011-2013 for China). Memory function was measured by 20-point immediate and delayed word recall summary scores over a subsequent 5- or 6-year follow-up period. Within each country, we used sampling-weighted, multivariable-adjusted causal mediation analysis (product method) to estimate the total and direct effects of non-mortgage debts on memory function, and the indirect effects mediated by depressive symptoms and dissatisfaction with life. We observed significant mediating effects of depressive symptoms on the association between non-mortgage debts and memory function in China only. Dissatisfaction with life had a significant mediating effect on this association in US and China, not in England. Although more investigation is needed, the relationships between non-mortgage debts and later-life memory function through psychosocial pathways may differ across macro-level socioeconomic structures.

